# High Rates of Antimicrobial Resistance and Emergence of High‐Risk Clones in Community‐Acquired Uropathogenic *Escherichia coli*

**DOI:** 10.1002/mbo3.70074

**Published:** 2025-10-21

**Authors:** Vívian Santos Galvão, Adriano Souza Santos Monteiro, João Lucas Pinheiro Leite, Isabela Oliveira Sousa, Soraia Machado Cordeiro, Joice Neves Reis

**Affiliations:** ^1^ Postgraduate Program in Pharmacy, Faculty of Pharmacy Federal University of Bahia Salvador Bahia Brazil; ^2^ Faculty of Pharmacy Federal University of Bahia Salvador Bahia Brazil; ^3^ Postgraduate Program in Microbiology Federal University of Bahia Salvador Bahia Brazil

**Keywords:** antimicrobial resistance, community‐acquired Infections, high‐risk clones, uropathogenic *Escherichia coli*, virulence markers

## Abstract

Urinary tract infection (UTI) is the most common bacterial infection globally and is often treated empirically in community settings, contributing to antimicrobial resistance. Uropathogenic *E. coli* (UPEC) is the leading cause of community‐acquired UTIs (CA‐UTIs), yet data on its clinical and molecular characteristics remain limited. To investigate the clinical and microbiological features of CA‐UTIs, focusing on antimicrobial resistance, phylogenetic groups, virulence genes, and clonal profiles. Randomly selected *E. coli* isolates from CA‐UTI cases underwent antimicrobial susceptibility testing. PCR was used to detect β‐lactamase genes, phylogenetic groups, and key virulence factors. MLST was used for clonal typing. Clinical and demographic data were obtained through structured interviews. Among 98 CA‐UPEC isolates, most were from female patients (95.9%), median age 48 years. High resistance was observed to ampicillin (50.0%), trimethoprim‐sulfamethoxazole (34.7%), and fluoroquinolones (33.7%), while resistance to nitrofurantoin and fosfomycin was low (1%). Multidrug resistant (MDR) strains accounted for 24.5%, with 6.1% being ESBL producers, all harboring *bla*_CTX‐M‐type_ genes. Phylogenetic group B2 (48.0%) carried the most virulence genes, while group A had more MDR strains. Common virulence genes included *fimH* (95.9%), *PAI* IV_536_ (77.6%), and *fyuA* (76.5%). Genes *aer*, and *iutA* were linked to MDR, while *papC, sfa, hlyA* were more common in non‐MDR isolates. ST1193, a high‐risk clone, was the most prevalent (25.0%), found only among MDR strains. ST73 and ST127 were associated with multiple virulence genes but were non‐MDR. Nitrofurantoin and fosfomycin remain effective options for CA‐UTIs. Continuous surveillance is vital to guide treatment and track resistance trends.

## Graphical abstract

A total of 98 *E. coli* isolates from community‐acquired UTIs were analyzed, revealing high resistance rates to trimethoprim‐sulfamethoxazole and fluoroquinolones. Phylogenetic group B2 was the most prevalent and associated with increased virulence, while ST1193 emerged as the dominant MDR clone. These findings underscore the need for ongoing community surveillance and cautious use of these antimicrobials, as well as the importance of regional monitoring to guide treatment strategies.

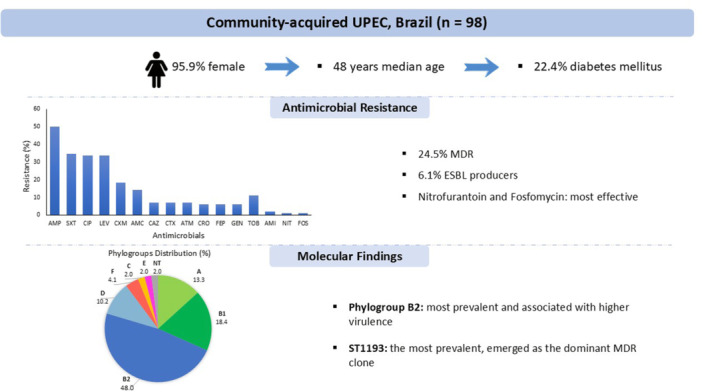

## 1 Introduction

Urinary tract infections (UTIs) are among the most prevalent bacterial infections globally, affecting over 150 million individuals yearly and imposing significant social and economic burdens (He et al. 2025). Community‐acquired UTIs (CA‐UTIs) constitute the majority of these cases, with uropathogenic *Escherichia coli* (UPEC) responsible for approximately 70–80% of infections in otherwise healthy individuals (Flores‐Mireles et al. 2015; Tano et al. 2022). In Brazil, however, most investigations on UPEC have been concentrated in the South and Southeast regions, and well‐characterized studies remain scarce, limiting a comprehensive understanding of the national epidemiological scenario.

Managing UTIs has become increasingly challenging due to the rapid emergence and dissemination of antibiotic‐resistant *E. coli* strains. Notably, extended‐spectrum β‐lactamase (ESBL)‐producing *E. coli* has been identified as the second most critical priority pathogen for public health, with a growing prevalence not only in hospital settings but also within the community (Sati et al. 2025; Fuga et al. 2022). Additionally, resistance to ESBLs, fluoroquinolones, and aminoglycosides severely restricts therapeutic options, heightens the risk of treatment failure, and often necessitates the use of last‐resort antibiotics such as carbapenems (Naghavi et al. 2024; Zeng et al. 2022).

UPEC strains originate from various phylogenetic groups, each associated with different virulence profiles and antibiotic resistance patterns. The pathogenicity of *E. coli* is primarily attributed to a multitude of virulence factors—such as adhesins, iron acquisition systems, and toxins—that facilitate colonization, invasion, immune evasion, and tissue damage during UTIs (Bien et al. 2012; Foxman 2014; Barrios‐Villa et al. 2023). These virulence genes are often located on pathogenicity‐associated islands (PAIs), which can be horizontally transferred between strains, enhancing UPEC's adaptability and pathogenic potential (Nascimento et al. 2022; Desvaux et al. 2020).

Epidemiological studies have revealed that a limited number of pandemic sequence types (STs) are responsible for most ExPEC infections worldwide. Specifically, ST131, ST69, ST73, ST95, ST127, ST10, and ST38 have been identified as predominant lineages, collectively accounting for over 50% of ExPEC infections globally (Manges et al. 2019; Fibke et al. 2019; Yamaji et al. 2018). These lineages are often associated with multidrug resistance and/or heightened virulence, contributing significantly to the global burden of UTIs. However, data on the distribution of these CA‐UPEC lineages in Brazil remain scarce. Thus, this study aims to characterize UPEC isolates from CA‐UTIs, focusing on their antibiotic resistance profiles, phylogenetic groups, virulence factors, and clonal lineages to provide insights into their pathogenic potential and support targeted strategies for effective UTI management.

## 2 Material and Methods

### 2.1 Study Design and Recruitment Process

This study included samples of patients with CA‐UTI obtained from a cross‐sectional study conducted from April 2019 to January 2022 at the Clinical Analysis Laboratory of the School of Pharmacy at the Federal University of Bahia, Brazil. Patients suspected of having a UTI with a request for urine culture were included when *E. coli* was identified in a positive urine culture, without age or sex restrictions. A new isolate was considered if a patient had a positive urine culture for *E. coli* more than 90 days after a previous positive result.

To classify an infection as a CA, patients must not have received intravenous therapy, renal dialysis, invasive urinary procedures, or any specialized or wound care within the previous 30 days. Additionally, they must not have undergone surgery or been hospitalized within the past 90 days nor resided in nursing homes or long‐term care facilities in the 12 months before urine culture collection. Information on sex, age, symptoms, and other relevant clinical and demographic data were gathered through questionnaire interviews.

### 2.2 Bacterial Identification and Antimicrobial Resistance Profile

A positive urine culture result was defined as growth ≥ 10^5^ CFU/mL, and *E. coli* isolates were identified through standard biochemical tests.

Antimicrobial susceptibility testing was conducted using the Kirby‐Bauer disk diffusion method for 19 antibiotics, including ampicillin (10 μg), amoxicillin‐clavulanate (20/10 μg), piperacillin‐tazobactam (100/10 μg), cefepime (30 μg), cefotaxime (30 μg), ceftriaxone (30 μg), ceftazidime (30 μg), cefuroxime (30 μg), aztreonam (30 μg), imipenem (10 μg), meropenem (10 μg), gentamicin (10 μg), tobramycin (10 μg), amikacin (30 μg), ciprofloxacin (5 μg), levofloxacin (5 μg), trimethoprim‐sulfamethoxazole (1.25/23.75 μg), fosfomycin (200 μg) and nitrofurantoin (300 μg), according to Clinical and Laboratory Standards Institute guidelines, 2023 (CLSI 2023). ESBL production was investigated using a disk approximation test, and isolates resistant to at least three different antimicrobial categories were classified as multidrug‐resistant (MDR) (Jiménez Pearson et al. 2019; Magiorakos et al. 2012). Quality control was ensured using *E. coli* ATCC 25922.

### 2.3 β‐lactamases Genes

DNA extraction from the isolates was performed using the boiling method, followed by PCR amplification to detect β‐lactamase genes, including *bla*_SHV_, *bla*_TEM_, *bla*_OXA‐1‐like_, *bla*_CTX‐M‐1_, *bla*_CTX‐M‐2_, *bla*_CTX‐M‐9_, *bla*_IMP_, *bla*_VIM_, *bla*_KPC_ according to the protocol described by (Dallenne et al. 2010). Additionally, the presence of *bla*_CTX‐M‐type_ genes was assessed following the method described by (Pagani et al. 2003). PCR products were visualized on 2% agarose gels stained with GelRed after electrophoresis, and images were captured using the l‐Pix EX system (Loccus).

### 2.4 Phylogeny Classification

The phylogenetic groups were determined by PCR assay, using a set of DNA markers to classify isolates into the eight recognized groups (A, B1, B2, C, D, E, F, G) and clade I, as previously described by Clermont and colleagues (Clermont et al. 2019, 2013).

### 2.5 Virulence Genes and Pathogenicity Islands

PCR assays were also used to identify key virulence factor genes: *hlyA* (hemolysin), *cnf‐*1 (cytotoxic necrotizing factor), *fimH* (type 1 fimbrial adhesin), *papC*, and *papG* (P‐fimbriae), *sfa* (S‐fimbrial adhesin), *draD*/*afa* (afimbrial adhesin), *traT* (serum resistance‐associated outer membrane lipoprotein), *kpsM* and *neuA* (capsules) and siderophore: *iutA* and *aer* (aerobactin), *fyuA* (yersiniabactin), *iroN* (salmochelina) (Yamamoto et al. 1995; Poey et al. 2012). To detect major pathogenicity islands (PAIs), a triplex PCR was performed, including PAI I_CFT076_, PAI II_CFT076_, and PAI IV_536_ (Johnson and Stell 2000; Sabaté et al. 2006). The oligonucleotides used for the 15 virulence genes and PAIs are detailed in Appendices (Table A1). Positive and negative control strains were included in each assay.

A virulence score (VS) was assigned to assess the virulence of the strains, with one point awarded for each detected gene, with adjustment for multiple detections of the *aer* and/or *iutA*.

### 2.6 Genotyping by Multilocus Sequence Typing

Multilocus sequence typing (MLST) was performed on all isolates classified as MDR and on those harboring 10 or more virulence genes, using this threshold as a pragmatic strategy to prioritize isolates with a higher predicted pathogenic potential, given resource limitations. The *E. coli* MLST scheme developed by Achtman, based on sequencing internal fragments of seven housekeeping genes (*adk, fumC, gyrB, icd, mdh, purA*, and *recA*) was applied (Wirth et al. 2006). Each isolate's sequence type (ST) was determined using the *Escherichia* spp. PubMLST database (https://pubmlst.org/organisms/escherichia-spp), and data was analyzed using the goeBURST algorithm implemented in the PHYLOViZ program (http://www.phyloviz.net/goeburst/). Clusters were defined as groups of STs sharing five or six identical loci, including the founder ST and its single‐ or double‐locus variants (SLVs or DLVs).

### 2.7 Statistical Analysis

Associations between UPEC phylogroups, antimicrobial resistance, and the presence of virulence factors were initially assessed using Fisher's exact test for categorical variables and the Mann‐Whitney U test for continuous variables. Binomial logistic regression models were applied to evaluate the role of virulence genes and phylogenetic background as predictors of resistance to specific antimicrobial agents. Multivariate models were constructed to adjust for potential confounders and to account for multiple comparisons. Statistical significance was set at *p* < 0.05. All analyzes were performed using RStudio software (version 2025.09.0 + 387).

## 3 Results

### 3.1 Clinical Characteristics

During the study period, 4,099 urine cultures were performed, of which 451 (11.0%) yielded positive results. Among the positive cultures, *E. coli* was the most frequently isolated pathogen, accounting for 60.5% (282/451), followed by *Klebsiella pneumoniae* (13.1%; 59/451), *Streptococcus agalactiae* (10.6%; 48/451), *Staphyloccocus saprophyticus* (2.4%; 11/451), *Klebsiella aerogenes* (2.2%–10/451), and *Citrobacter* sp. (1.3%; 6/451). Other microorganisms accounted for ≤ 1% each.

Ninety‐five patients with CA‐UTI caused by *E. coli* consented to participate in the study. One patient experienced two distinct UTIs during the study period, and another experienced three, resulting in 98 *E. coli* isolates. The majority of infections occurred in female patients (95.9%; 94/98), with a median age of 48 years (range: 31‐65) (Table 1). The most frequently reported comorbidity was diabetes mellitus (22.4%; 22/98). The primary clinical symptoms included burning micturition (50.0%; 49/98) and dysuria (41.8%; 41/98). All patients who reported the combined symptoms of burning micturition, dysuria, and fever also reported urinary incontinence.

**Table 1:** Demographic and clinical characteristics of patients with community‐acquired urinary tract infections caused by *E. coli* (*n* = 98), with comparison of B2 versus non‐B2 phylogroups.

Characteristics	CA‐UTI 98 (%)	B2	Non‐B2	*p* value	Univariate	
		47 (%)	51 (%)		OR	95% CI
**Demographic data**						
Female	94 (95.9)	44 (93.6)	50 (98.0)	0.35	—	—
Male	4 (4.1)	3 (6.4)	1 (2.0)		3.41	0.42–70.3
Pregnant	15 (16.0)	9 (19.1)	6 (11.8)	0.40	1.78	0.59–5.73
Age (years), median (1 qt–3qt)^a^	48 (32‐65)	48 (31‐66)	46 (30‐63)	0.97		
< 30	21 (21.4)	10 (21.2)	11 (21.6)		—	—
30–60	42 (42.9)	19 (40.4)	23 (45.1)		0.96	0.34–2.75
> 60	31 (31.6)	15 (31.9)	16 (31.4)		1.10	0.36–3.35
**Comorbidities**						
Diabetes mellitus	22 (22.4)	12 (25.5)	10 (19.6)	0.63	1.41	0.54–3.71
Rheumatoid arthritis	6 (6.1)	1 (2.1)	5 (9.8)	0.21	0.20	0.01–1.30
Systemic arterial hypertension	31 (31.6)	15 (31.9)	16 (31.4)	> 0.99	1.03	0.43–2.41
**Symptoms**						
BM + DYS	29 (29.6)	12 (25.5)	17 (33.3)	0.51	0.69	0.28–1.64
UI + DYS	24 (24.5)	11 (23.4)	13 (25.5)	> 0.99	0.89	0.35–2.25
BM + UI	23 (23.5)	9 (19.1)	14 (27.4)	0.35	0.63	0.23–1.60
BM + DYS + UI	18 (18.4)	7 (14.9)	11 (21.6)	0.44	0.64	0.21–1.78
BM + DYS + UI + FEV	4 (4.1)	2 (4.2)	2 (3.9)	> 0.99	1.09	0.13–9.38
**Multidrug resistance**				> 0.99		
Non‐MDR	74 (75.5)	35 (74.5)	39 (76.5)		—	—
MDR	24 (24.5)	12 (25.5)	12 (23.5)		1.11	0.44–2.82

Abbreviations: BM, burning micturition; CA, community‐acquired; CI, confidence interval; DYS, dysuria (painful urination); FEV, fever; MDR, multidrug‐resistant; OR, odds ratio; qt, quartile; UI, urinary incontinence; UTI, urinary tract infection.

^a^ Mann‐Whitney U test; other comparisons were performed using Fisher's exact test.

### 3.2 Antimicrobial Resistance

**Figure 1:**
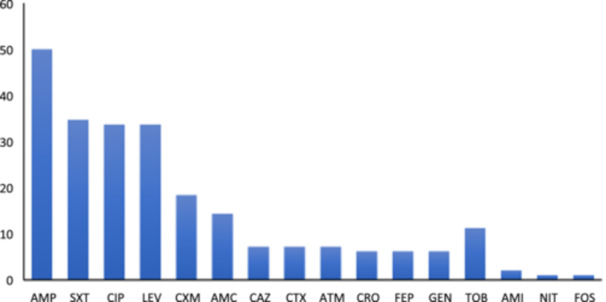
Frequency of antimicrobial resistance among 98 *Escherichia coli* isolates from community‐acquired urinary tract infections in Salvador, Brazil. AMI, amikacin; AMC, amoxicillin‐clavulanate; AMP, ampicillin; ATM, aztreonam; CAZ, ceftazidime; CIP, ciprofloxacin; CRO, ceftriaxone; CTX, cefotaxime; CXM, cefuroxime; FEP, cefepime; FOS, fosfomycin; GEN, gentamicin; LEV, levofloxacin; NIT, nitrofurantoin; SXT, trimethoprim‐sulfamethoxazole; TOB, tobramycin.

A notable proportion of *E. coli* isolates exhibited resistance to antibiotics commonly prescribed for CA‐UTI, such as ampicillin (50.0%), trimethoprim‐sulfamethoxazole (34.7%), fluoroquinolones (33.7%), cefuroxime (18.4%) and amoxicillin‐clavulanate (14.3%). Lower resistance rates were detected for aztreonam, third‐ and fourth‐generation cephalosporins, gentamicin, and amikacin (Figure 1). Only one isolate was non‐susceptible to nitrofurantoin, and another was fosfomycin. No resistance was identified to piperacillin‐tazobactam, imipenem, or meropenem. Overall, 70.4% of the isolates were resistant to at least one agent, while the remainder showed susceptibility to all tested antimicrobials.

MDR profile was observed in 24.5% (24/98) of the isolates, and 6.1% (6/98) were identified as ESBL producers. Additionally, the median age of patients with MDR isolates (54 years) and ESBL‐producing isolates (58 years) was higher than that of patients with non‐MDR isolates (45 years). However, the difference was not statistically significant.

We also analyzed the presence of β‐lactamase genes and found that 33 out of 98 isolates were positive. Among these, 25 harbored *bla*_TEM_, one carried only *bla*_CTX‐M‐1_, two carried only *bla*_CTX‐M‐9_, and five exhibited coexistences, including combinations such as *bla*_TEM_ + *bla*_CTX‐M‐1_; *bla*_TEM_ + *bla*_CTX‐M‐2_; *bla*_TEM_ + *bla*_CTX‐M‐9_; *bla*_TEM_ + *bla*_OXA‐1‐like_, and *bla*_TEM_ + *bla*_CTX‐M‐9_ + *bla*_OXA‐1‐like_. All ESBL‐producing *E. coli* isolates carried a *bla*_CTX‐M_ gene.

### 3.3 Phylogenetic Group Distribution

Phylogroup B2 was the most prevalent among the UPEC isolates, comprising 48.0% (47/98) of the strains, followed by B1 (18.4%; 18/98), A (13.3%; 13/98), and D (10.2%; 10/98). Less common phylogroups included F (4.1%; 4/98), C (2.0%; 2/98), and E (2.0%; 2/98). Based on the Clermont method, two isolates could not be assigned to any phylogroup. A statistically significant association was observed between phylogroup A and multidrug‐resistant phenotype (*p* = 0.01) (Table 2).

**Table 2:** Distribution of *E. coli* isolates from community‐acquired urinary tract infections according to phylogenetic groups and multidrug‐resistant phenotype.

Phylogroup	MDR *n* = 24 (%)	Non‐MDR *n* = 74 (%)	Total *n* = 98	*p* value^a^	Univariate OR 95% CI	
A	7 (29.2)	6 (8.1)	13 (13.3)	**0.01**	4.67	1.38–16.3
B1	2 (8.3)	16 (21.6)	18 (18.4)	0.16	0.33	0.05–1.29
B2	12 (50.0)	35 (47.3)	47 (48.0)	0.82	1.11	0.44–2.82
D	2 (8.3)	8 (10.8)	10 (10.2)	0.73	0.75	0.11–3.28
Others*	1 (4.2)	9 (12.2)	10 (10.2)	0.28	0.31	0.02–1.81

*Note:* Bold values are indicate statistically significance.

Abbreviations: CI, confidence interval; MDR, multidrug‐resistant; OR, odds ratio.

* Includes phylogroups C, E, F, and non‐typeable.

^a^ Fisher's exact test.

### 3.4 Virulence Genes Distribution and Association With Phylogroups

Table 3 summarize the prevalence of virulence gene among *E. coli* isolates. The adhesin gene *fimH* was the most frequently detected (95.9%; 94/98), followed by the siderophore gene *fyuA* (76.5%; 75/98) and the capsule synthesis gene *kpsM* (57.1%; 56/98). Although less prevalent overall, the virulence genes *papG, cnf‐1*, *sfa*, and *hlyA* were almost exclusively found in isolates belonging to phylogroup B2. Additionally, genes such as *neuA*, *kpsM*, *fyuA*, and pathogenicity islands (PAI I_CFT073_, PAI II_CFT073_, and PAI IV_536_) were significantly associated with phylogroup B2 (*p* < 0.05) (Supporting Information Table A2).

**Table 3:** Distribution of virulence genes by phylogenetic groups of *E. coli* isolates from community‐acquired urinary tract infections (*n* = 98).

Virulence genes and PAIs	Distribution of phylogenetic groups								
	A (*n* = 13)	B1 (*n* = 18)	B2 (*n* = 47)	D (*n* = 10)	C (*n* = 2)	E (*n* = 2)	F (*n* = 4)	NT (*n* = 2)	Total (*n* = 98)
*papG*			5						5
*cnf1*			8						8
*sfa*			12						12
*hlyA*			12	1					13
*draD/afa*	2		1	1					4
*iroN*	3		13	2	1				19
*papC*		1	12	2			4	2	21
*traT*	7	3	11	3	1		1	2	28
*neuA*	1	1	21	1	1	1	4		30
*iutA*	5	2	23	7	1		3	2	44
*aer*	6	3	23	6	1		4	2	45
*kpsM*	1		43	8			4		56
*fyuA*	6	7	46	9	1		4	2	75
*fimH*	12	17	47	8	2	2	4	2	94
PAI I_CFT073_	1		18				2		21
PAI II_CFT073_			27	2					29
PAI IV_536_	7	7	46	9	1		4	2	76

Abbreviations: NT, non‐typeable; PAI, pathogenicity‐associated island.

Phylogroup B2 exhibited the highest proportion of isolates carrying multiple virulence factors. Notably, all isolates harboring 10 or more virulence genes were members of this group. However, when considering the average number of virulence genes per isolate, phylogroup F, despite being infrequent, showed the highest virulence score (7.5 genes/isolate), followed closely by B2 (7.3 genes/isolate) (Supporting Information Table A3). In contrast, phylogroups A and B1, traditionally regarded as commensal, displayed lower virulence score (3.6 and 2.1 genes/isolate, respectively), yet together accounted for more than 30% of all CA‐UTI cases in this study.

The strains carrying a higher number of virulence genes, defined by a virulence score ≥ 10 and representing 13.5% (12/98) of the isolates, were exclusively found within phylogroup B2. Interestingly, these isolates demonstrated an inverse relationship with antimicrobial resistance: none were classified as MDR, and 83.3% (10/12) lacked any of the tested resistance genes.

When analyzing the relationship between virulence factors and antimicrobial resistance, isolates carrying *sfa*, *hlyA*, and *papC* were significantly more susceptible to ciprofloxacin and levofloxacin. Additionally, the presence of *papC* was strongly associated with non‐MDR isolates (Table 4). On the other hand, isolates harboring the siderophore genes *iutA* and *aer* were significantly associated with resistance to ampicillin, ciprofloxacin, levofloxacin, and the MDR phenotype (*p* < 0.05).

**Table 4:** Association between virulence genes, antibiotic resistance, and multidrug‐resistant phenotypes in community‐acquired uropathogenic *E. coli* strains (*n* = 98).

Antibiotics	Trait^a^	Prevalence of trait among isolates (*n* [%])		*p* value^b^	Univariate	
		Susceptible	Resistant		OR	95% CI
Ampicillin	*iutA*	15/49 (30.6)	29/49 (59.2)	< 0.01	2.97	1.30–6.79
	*aer*	17/49 (34.7)	28/49 (57.1)	0.04	2.28	1.01–5.14
Ciprofloxacin/Levofloxacin	*sfa*	12/65 (18.5)	0/33 (0.0)	< 0.01	0.06	0.00–1.25
	*hlyA*	12/65 (18.5)	1/33 (3.0)	0.05	0.20	0.03–1.21
	*papC*	19/65 (29.2)	2/33 (6.1)	< 0.01	0.19	0.05–0.78
	*neuA*	12/65 (18.5)	18/33 (54.5)	< 0.01	5.11	2.02–12.9
	*iutA*	21/65 (32.3)	23/33 (69.7)	< 0.01	4.63	1.88–11.4
	*aer*	22/65 (33.8)	23/33 (69.7)	< 0.01	4.33	1.76–10.6
Trimethoprim + Sulfamethoxazole	*iutA*	23/64 (35.9)	21/34 (61.8)	0.02	2.81	1.19–6.62
		**Non‐MDR**	**MDR**			
Mutidrug Resistance	*sfa*	12/74 (16.2)	0/24 (0.0)	0.03	9.80	0.50–193
	*hlyA*	13/74 (17.6)	0/24 (0.0)	0.03	10.8	0.55–209
	*papC*	20/74 (27.0)	1/24 (1.3)	0.02	5.89	1.01–34.4
	*iutA*	27/74 (36.5)	17/24 (70.8)	< 0.01	0.25	0.09–0.66
	*aer*	28/74 (37.8)	17/24 (70.8)	< 0.01	0.26	0.10–0.70

Abbreviations: CI, confidence interval; MDR, multidrug‐resistant; OR, odds ratio.

^a^ Only genes exhibiting statistically significant associations to the tested antibiotics are shown.

^b^ Fisher's exact test.

### 3.5 MLST Analysis and Clonal Distribution

MLST analysis of 36 isolates identified 16 distinct sequence type (STs). The most prevalent was ST1193 (25.0%; 9/36), with all isolates exhibiting fluoroquinolone resistance and belonging to phylogroup B2. ST73 and ST127 were the next most frequent, each accounting for four isolates (11.1%).

GoeBURST analysis, based on single‐locus variants (SLVs) or double‐locus variants (DLVs), revealed two main clonal complexes. Clonal complex CC14 comprises ST1193 and ST14, representing 10 isolates (27.8%). Clonal complex CC10 included six isolates (16.7%) distributed across five STs, ST10 (*n* = 2), ST44, ST617, ST744, and ST1286 (each *n* = 1). The remaining STs were singletons, showing no clonal relatedness within the analyzed data set.

MDR isolates were distributed across several STs, including ST1193, ST131, ST10, ST224, ST38, ST44, ST46, ST410, ST617, ST744, and ST1286. Most MDR isolates (87.5%; 21/24) belonged to high‐risk pandemic clones associated with human infections. In contrast, the isolates harboring the highest virulence factors were ST73, ST127, ST698, ST95, and ST14, all of which were non‐MDR.

## 4 Discussion

In this study, *E. coli* was the most frequently isolated pathogen in community‐acquired urinary tract infections (CA‐UTI). More than 90% of cases occurred in women, with the highest frequency among individuals aged 31‐59 years, aligning with previous studies from Brazil and Japan (Tano et al. 2022; Matsukawa et al. 2019). Diabetes mellitus was the most common comorbidity, consistent with its well‐established role as a major risk factor for UTIs. These findings align with early CA‐UTI studies reporting diabetes prevalence rates between 21% and 31% (Foxman 2014; Blanco et al. 2016; Bian et al. 2024).

Phylogenetic analysis showed that most UPEC isolates belonged to phylogroup B2 (48.0%), followed by B1 (18.4%) and A (13.3%). This distribution is consistent with findings from Egypt (El‐baz et al. 2022) and Romania (Cristea et al. 2019), but differs from reports from other regions of Brazil, where higher frequencies of phylogroups B2 and D and lower proportions of A and B1, have been observed (Lara et al. 2017; de Souza da‐Silva et al. 2020; De Souza. 2019; Coura et al. 2021). Notably, several studies highlight that phylogroup distribution can vary significantly, even within the same country or geographic region, reflecting substantial heterogeneity across different populations (El‐baz et al. 2022; Rezatofighi et al. 2021; Wang et al. 2023; Miranda‐Estrada et al. 2017).

Our findings highlight that strains traditionally considered commensal, particularly those belonging to phylogroups A and B1, which accounted for approximately one‐third of the isolates, are capable of causing extraintestinal infections. This ability is attributed to *E. coli*'s remarkable capacity to acquire virulence genes through horizontal gene transfer within the gastrointestinal tract (Sabaté et al. 2006; Lara et al. 2017). Acquiring these genes enhance the pathogen's virulence potential, facilitating effective colonization and infection of the urinary tract.

The antimicrobial resistance patterns observed in this study reinforce the recommendation of nitrofurantoin and fosfomycin as first‐line treatments for CA‐UTIs. These findings are consistent with reports from other regions of Brazil (Tano et al. 2022; de Souza da‐Silva et al. 2020; De Souza. 2019; Coura et al. 2021), as well as from Europe (Cristea et al. 2019; Grados et al. 2019) and the Middle East (Yazdanpour et al. 2020; Darraj 2023). Our results underscore antimicrobial stewardship's importance in mitigating the risk of increasing resistance, particularly nitrofurantoin. While fosfomycin remains underutilized, primarily due to its relatively high cost and unavailability in the public health system, nitrofurantoin is more commonly prescribed. Furthermore, our findings highlight the need for caution when prescribing fluoroquinolones and trimethoprim‐sulfamethoxazole, given that resistance rates exceeded 30% among isolates despite continued use in CA‐UTI treatment.

The frequencies of MDR phenotype and ESBL‐producing strains among CA‐UPEC isolates in our study are comparable to those reported in other Brazilian studies (Tano et al. 2022; de Souza da‐Silva et al. 2020; De Souza. 2019; Coura et al. 2021). In contrast, significantly higher resistance rates have been observed in Mexico and Iran, where the prevalence of MDR and ESBL‐producing strains exceeds 50% (Miranda‐Estrada et al. 2017; Belmont‐Monroy et al. 2022; Mostaghimi et al. 2024). Our analysis, revealed that MDR isolates were significantly associated with phylogroup A, which differs from findings in Rio de Janeiro, Brazil, where MDR strains were predominantly linked to phylogroups B2, D, and F (Campos et al. 2018).

Moreover, the most recent study conducted in our region indicated that the prevalence of ESBL‐producing *E. coli* was less than one percent (Berman et al. 2014). This highlights a concerning increase in resistance over the past decade in CA‐UTI cases. This rising trend is alarming and corresponds with global reports of increasing antimicrobial resistance in community settings (Achukutty et al. 2020; Devi et al. 2020). This pattern has been particularly evident in several Asian countries (Kumar et al. 2021; Jia et al. 2021; Islam et al. 2022; Halaji et al. 2021) and may have been further exacerbated by the widespread overuse and misuse of antibiotics during the COVID‐19 pandemic (Venugopal et al. 2024; Abdelaziz Abdelmoneim et al. 2024).

UPEC strains harbor a wide array of virulence factors that enable them to effectively colonize the urinary tract and evade the host immune defenses (Barrios‐Villa et al. 2023; Etefia 2021). In the present study, all isolates were positive for at least two of the virulence genes analyzed. The most prevalent was *fimH*, a critical component of UPEC pathogenicity, as it mediates adhesion to uroepithelial cells (Hyun et al. 2021; Khairy et al. 2019). The *fimH* gene has emerged as a promising vaccine targed for UTI prevention, primarily due to the ability of specific antibodies to inhibit its function and block bladder colonization (Flores‐Mireles et al. 2015). The iron acquisition system encoded by *fyuA* and PAI IV_536_ was frequently detected among UPEC strains in CA‐UTI, with prevalence rates of 76.5% and 77.5% among UPEC strains, respectively. Similar findings have been reported in other studies, as the expression of this factor plays a critical role in the pathogenesis of ascending UTIs and is essential to overcoming iron limitation in host environments such as urine (Nascimento et al. 2022; Etefia 2021).

Interestingly, we observed an inverse relationship between antibiotic resistance and virulence. Phylogroups F and B2 exhibited higher virulence profiles but were predominantly non‐MDR, in contrast to findings from other studies that report a positive correlation between virulence and resistance (Campos et al. 2018; Allami et al. 2022; Ballesteros‐Monrreal et al. 2020).

Regarding molecular genotyping, MLST analysis revealed ST1193 as the predominant clone among MDR CA‐UPEC isolates in our study, consistent with findings from a study in China (Zeng et al. 2021). In contrast, studies from the USA, Canada, and Europe have reported lower frequencies of ST1193; however, its global dissemination has been well documented in UPEC and other ExPEC isolates across multiple continents (Fibke et al. 2019; Yamaji et al. 2018; Flament‐Simon et al. 2020; Tsui et al. 2024). In Brazil, previous studies, particularly from Rio de Janeiro, have reported ST69, ST73, and ST131 as the most prevalent CA‐UPEC lineages, and a nationwide study employing a One Health approach highlighted the clonal heterogeneity of UPEC across different Brazilian regions but did not report the presence of ST1193, suggesting its emergence as a novel clone in our region (Fuga et al. 2022; de Souza da‐Silva et al. 2020).

ST1193 is recognized as an emerging clone, and our findings emphasize its growing relevance in MDR community‐associated isolates. Pitout et al (Pitout et al. 2022). suggest that ST1193 follows a similar evolutionary trajectory to *E. coli* ST131, often described as “the most successful MDR clone of all time” due to shared resistance traits, high transmissibility, and increasing global dissemination. Importantly, most MDR isolates in our study (87.5%; 21/24) belonged to high‐risk pandemic clonal groups in humans, reinforcing the critical role of epidemic clones in spreading of antimicrobial resistance.

This study has some limitations, most notably its local scope, which may restrict the generalizability of the findings. Recruitment, was also constrained by logistical challenges, resulting in the inclusion of only a subset of eligible cases. While MLST provided important insights into clonal distribution, the absence of whole genome sequencing as well as plasmid and integron analysis limited a deeper exploration of the genetic determinants of resistance and virulence. Even so, the resistance patterns observed and the emergence of high‐risk clones, such as ST1193, mirror the global trends, indicating broader epidemiological relevance. Furthermore, the scarcity of published data from our region, together with the inclusion of well‐characterized community‐acquired infections supported by detailed clinical data, underscores the relevance of this study.

In conclusion, *E. coli* was responsible for most CA‐UTI cases in this study. Phylogroup B2, characterized by an extensive repertoire of virulence genes, and phylogroup A, associated with greater antimicrobial resistance, were the most prevalent. Among the antibiotics tested, nitrofurantoin and fosfomycin demonstrated the highest efficacy against UPEC isolates in our study. In contrast, high resistance rates to commonly prescribed empirical therapies, such as ampicillin, fluoroquinolones, and trimethoprim‐sulfamethoxazole, highlight the need for updated treatment guidelines based on local resistance patterns. Notably, ST1193 emerged as the dominant MDR clone, and its increasing presence in community settings reinforces the urgent need for integrated surveillance and infection control strategies to curb the spread of MDR *E. coli*.

## Author Contributions

**Vivian Santos Galvão:** conceptualization (supporting), investigation (lead), methodology (supporting), visualization (lead), data curation (lead), writing – original draft (lead), validation (lead), formal analysis (lead), writing – review and editing (equal). **Adriano Souza Santos Monteiroand** and **João Lucas Pinheiro Leite:** investigation (supporting), methodology (supporting), formal analysis (supporting), data curation (supporting), validation (supporting), writing – review and editing (equal). **Isabela Oliveira Sousa:** investigation (supporting), methodology (lead), validation (supporting), writing – review and editing (equal). **Soraia Machado Cordeiro:** supervision (supporting), conceptualization (supporting), project administration (supporting), resources investigation (supporting), funding acquisition (supporting), methodology (supporting), validation (supporting), writing – review and editing (equal). **Joice Neves Reis:** supervision (lead), conceptualization (lead), project administration (lead), resources investigation (supporting), funding acquisition (lead), methodology (supporting), data curation (supporting), formal analysis (supporting), visualization (supporting), validation (supporting), writing – review and editing (equal). All authors read and approved the final article.

## Ethics Statement

The study was conducted in accordance with the ethical standards of both the institutional and national research committee and was approved by the Research Ethics Committee of the Federal University of Bahia (approval numbers 2.170.080/2017 and 2.012.382).

## Consent

Written or verbal informed consent was obtained from all participants before data collection.

## Conflicts of Interest

The authors declare no conflicts of interest.

## Supporting information


**Table A1**: Oligonucleotides for the identification of virulence factors and pathogenicity islands. **Table A2**: Virulence factors and islands of pathogenicity relation with phylogenetic groups of *E. coli* isolates from CA‐UTI (n=98). **Table A3**: Patterns of virulence genes in *E. coli* from community‐acquired urinary tract infections, stratified by phylogenetic groups.

## Data Availability

The data sets supporting the conclusions of this article are included within the article. The raw data can be made available to interested researchers by the authors of this article if requested.

## References

[mbo370074-bib-0001] Abdelaziz Abdelmoneim, S. , R. Mohamed Ghazy E. Anwar Sultan , M. A. Hassaan , and M. Anwar Mahgoub . 2024. “Antimicrobial Resistance Burden Pre and Post‐COVID‐19 Pandemic With Mapping the Multidrug Resistance in Egypt: A Comparative Cross‐Sectional Study.” Scientific Reports 14, no. 1: 7176. 38531847 10.1038/s41598-024-56254-4 PMC10966009

[mbo370074-bib-0002] Achukutty, T. , H. Aravind , D. Emil , R. Ravina , R. Lakshmi , and T. Appu . 2020. “Antimicrobial Susceptibility of Uropathogens and Prescribing Patterns in Hospital‐ and Community‐Acquired Urinary Tract Infections in a Tertiary Care Hospital.” Journal of Applied Pharmaceutical Science 10, no. 11: 050–058.

[mbo370074-bib-0003] Allami, M. , M. Bahreini , and M. R. Sharifmoghadam . 2022. “Antibiotic Resistance, Phylogenetic Typing, and Virulence Genes Profile Analysis of Uropathogenic *Escherichia coli* Isolated From Patients in Southern Iraq.” Journal of Applied Genetics 63, no. 2: 401–412. 35143031 10.1007/s13353-022-00683-2

[mbo370074-bib-0004] Ballesteros‐Monrreal, M. G. , M. M. P. Arenas‐Hernández , and Y. Enciso‐Martínez , et al. 2020. “Virulence and Resistance Determinants of Uropathogenic *Escherichia coli* Strains Isolated From Pregnant and Non‐Pregnant Women From Two States in Mexico.” Infection and Drug Resistance 13: 295–310. 32099421 10.2147/IDR.S226215 PMC6997036

[mbo370074-bib-0005] Barrios‐Villa, E. , L. R. Picón , R. B. Reynaga , and M. M. de la P. Arenas‐Hernández . 2023. “An Updated Overview on the Resistance and Virulence of UPEC.” In Em: Trending Topics in Escherichia coli Research, 249–276. Springer International Publishing.

[mbo370074-bib-0006] Belmont‐Monroy, L. , R. M. Ribas‐Aparicio , E. González‐Villalobos , et al. 2022. “Molecular Typification of *Escherichia coli* From Community‐Acquired Urinary Tract Infections in Mexico.” International Journal of Antimicrobial Agents 60, no. 4: 106667. 36038094 10.1016/j.ijantimicag.2022.106667

[mbo370074-bib-0007] Berman, H. , M. G. Barberino , E. D. Moreira , L. Riley , and J. N. Reis . 2014. “Distribution of Strain Type and Antimicrobial Susceptibility of *Escherichia coli* Isolates Causing Meningitis in a Large Urban Setting in Brazil.” Journal of Clinical Microbiology 52, no. 5: 1418–1422. 24523478 10.1128/JCM.03104-13 PMC3993653

[mbo370074-bib-0008] Bian, C. , Y. Zhu , X. Fang , et al. 2024. “Risk Factors and Economic Burden for Community‐Acquired Multidrug‐Resistant Organism‐Associated Urinary Tract Infections: A Retrospective Analysis.” Medicine 103, no. 21: 38248. 10.1097/MD.0000000000038248 PMC11124715 38788007

[mbo370074-bib-0009] Bien, J. , O. Sokolova , and P. Bozko . 2012. “Role of Uropathogenic *Escherichia coli* Virulence Factors in Development of Urinary Tract Infection and Kidney Damage.” International Journal of Nephrology 2012: 1–15. 10.1155/2012/681473 PMC3312279 22506110

[mbo370074-bib-0010] Blanco, V. M. , J. J. Maya , A. Correa , et al. 2016. “Prevalencia Y Factores De Riesgo Para Infecciones Del Tracto Urinario De Inicio En La Comunidad Causadas Por *Escherichia coli* Productor De Betalactamasas De Espectro Extendido En Colombia.” Enfermedades Infecciosas Y Microbiología Clínica 34, no. 9: 559–565. 26774256 10.1016/j.eimc.2015.11.017 PMC5061630

[mbo370074-bib-0011] Campos, A. C. C. , N. L. Andrade , M. Ferdous , et al. 2018. “Comprehensive Molecular Characterization of *Escherichia coli* Isolates From Urine Samples of Hospitalized Patients in Rio De Janeiro, Brazil.” Frontiers in Microbiology 9: 243. 29503639 10.3389/fmicb.2018.00243 PMC5821075

[mbo370074-bib-0012] Clermont, O. , J. K. Christenson , E. Denamur , and D. M. Gordon . 2013. “The Clermont *Escherichia coli* Phylo‐Typing Method Revisited: Improvement of Specificity and Detection of New Phylo‐Groups.” Environmental Microbiology Reports 5, no. 1: 58–65. 23757131 10.1111/1758-2229.12019

[mbo370074-bib-0013] Clermont, O. , O. V. A. Dixit , B. Vangchhia , et al. 2019. “Characterization and Rapid Identification of Phylogroup G in *Escherichia coli*, a Lineage With High Virulence and Antibiotic Resistance Potential.” Environmental Microbiology 21, no. 8: 3107–3117. 31188527 10.1111/1462-2920.14713

[mbo370074-bib-0014] CLSI . Performance Standards for Antimicrobial Susceptibility Testing. 33rd Ed. CLSI Supplement M100. Clinical and Laboratory Standards Institute. 2023.

[mbo370074-bib-0015] Coura, F. M. , V. M. S. Savini , R. G. C. Xavier , et al. 2021. “Virulence Genes Profile and Antimicrobial Susceptibility of Community‐Acquired Bacterial Urinary Tract Infections in a Brazilian Hospital.” Current Microbiology 78, no. 11: 3913–3923. 34522976 10.1007/s00284-021-02650-2

[mbo370074-bib-0016] Cristea, V. C. , I. Gheorghe , I. Czobor Barbu , et al. 2019. “Snapshot of Phylogenetic Groups, Virulence, and Resistance Markers in *Escherichia coli* Uropathogenic Strains Isolated From Outpatients With Urinary Tract Infections in Bucharest, Romania.” BioMed Research International 2019: 1–8. 10.1155/2019/5712371 PMC6545812 31236408

[mbo370074-bib-0017] Dallenne, C. , A. Da Costa , D. Decré , C. Favier , and G. Arlet . 2010. “Development of a Set of Multiplex PCR Assays for the Detection of Genes Encoding Important β‐lactamases in Enterobacteriaceae.” Journal of Antimicrobial Chemotherapy 65, no. 3: 490–495. 20071363 10.1093/jac/dkp498

[mbo370074-bib-0018] Darraj, M. A. 2023. “The Appropriateness of Empirical Antimicrobial Treatment of Uncomplicated Urinary Tract Infection in Adult Female Patients in Jazan Region, Saudi Arabia.” Clinics and Practice 13, no. 4: 743–752. 37489416 10.3390/clinpract13040067 PMC10366825

[mbo370074-bib-0019] Desvaux, M. , G. Dalmasso , R. Beyrouthy , N. Barnich , J. Delmas , and R. Bonnet . 2020. “Pathogenicity Factors of Genomic Islands in Intestinal and Extraintestinal Escherichia coli.” Frontiers in Microbiology 11: 2065. 33101219 10.3389/fmicb.2020.02065 PMC7545054

[mbo370074-bib-0020] Devi, L. , S. Broor , R. Rautela , S. Grover , A. Chakravarti , and D. Chattopadhya . 2020. “Increasing Prevalence of *Escherichia coli* and *Klebsiella pneumoniae* Producing CTX‐M‐Type Extended‐Spectrum Beta‐Lactamase, Carbapenemase, and NDM‐1 In Patients From a Rural Community With Community Acquired Infections: A 3‐year Study.” International Journal of Applied and Basic Medical Research 10, no. 3: 156. 33088736 10.4103/ijabmr.IJABMR_360_19 PMC7534723

[mbo370074-bib-0021] El‐baz, R. , H. S. Said , E. S. Abdelmegeed , and R. Barwa . 2022. “Characterization of Virulence Determinants and Phylogenetic Background of Multiple and Extensively Drug Resistant *Escherichia coli* Isolated From Different Clinical Sources in Egypt.” Applied Microbiology and Biotechnology 106, no. 3: 1279–1298. 35050388 10.1007/s00253-021-11740-x PMC8816750

[mbo370074-bib-0022] Etefia, E. Virulence Factors of Uropathogenic *Escherichia coli*. Em: *Escherichia coli* ‐ Old and New Insights. IntechOpen; 2021.

[mbo370074-bib-0023] Fibke, C. D. , M. A. Croxen , H. M. Geum , et al. 2019. “Genomic Epidemiology of Major Extraintestinal Pathogenic *Escherichia coli* Lineages Causing Urinary Tract Infections in Young Women Across Canada.” Open Forum Infectious Diseases 6, no. 11: ofz431. 31696141 10.1093/ofid/ofz431 PMC6824535

[mbo370074-bib-0024] Flament‐Simon, S. C. , M. H. Nicolas‐Chanoine , V. García , et al. 2020. “Clonal Structure, Virulence Factor‐Encoding Genes and Antibiotic Resistance of *Escherichia coli*, Causing Urinary Tract Infections and Other Extraintestinal Infections in Humans in Spain and France During 2016.” Antibiotics (USSR) 9, no. 4: 161. 10.3390/antibiotics9040161 PMC7235800 32260467

[mbo370074-bib-0025] Flores‐Mireles, A. L. , J. N. Walker , M. Caparon , and S. J. Hultgren . 2015. “Urinary tract infections: Epidemiology, Mechanisms of Infection and Treatment Options.” In Nature Reviews Microbiology (13, 269–284. Nature Publishing Group). 25853778 10.1038/nrmicro3432 PMC4457377

[mbo370074-bib-0026] Foxman, B. 2014. “Urinary Tract Infection Syndromes.” Infectious Disease Clinics of North America 28, no. 1: 1–13. 24484571 10.1016/j.idc.2013.09.003

[mbo370074-bib-0027] Fuga, B. , F. P. Sellera , L. Cerdeira , et al. 2022. “WHO Critical Priority *Escherichia coli* as One Health Challenge for a Post‐Pandemic Scenario: Genomic Surveillance and Analysis of Current Trends in Brazil.” Microbiology Spectrum 10, no. 2: e01256‐21. 10.1128/spectrum.01256-21 PMC8941879 35234515

[mbo370074-bib-0028] Grados, M. C. , I. J. Thuissard , and J. I. Alós . 2019. “Stratification by Demographic and Clinical Data of the Antibiotic Susceptibility of *Escherichia coli* From Urinary Tract Infections of the Community.” Atención Primaria 51, no. 8: 494–498. 30104087 10.1016/j.aprim.2018.06.004 PMC6837129

[mbo370074-bib-0029] Halaji, M. , S. Shahidi , B. Ataei , A. Atapour , A. Feizi , and S. A. Havaei . 2021. “Molecular Epidemiology of blaCTX‐M Gene‐Producing Uropathogenic *Escherichia coli* Among Iranian Kidney Transplant Patients: Clonal Dissemination of CC131 and CC10.” Annals of Clinical Microbiology and Antimicrobials 20, no. 1: 65. 34496873 10.1186/s12941-021-00470-7 PMC8424993

[mbo370074-bib-0030] He, Y. , J. Zhao , L. Wang , et al. 2025. “Epidemiological Trends and Predictions of Urinary Tract Infections in the Global Burden of Disease Study 2021.” Scientific Reports 15, no. 1: 4702. 39922870 10.1038/s41598-025-89240-5 PMC11807111

[mbo370074-bib-0031] Hyun, M. , J. Y. Lee , and H. Kim . 2021. “Differences of Virulence Factors, and Antimicrobial Susceptibility According to Phylogenetic Group in Uropathogenic *Escherichia coli* Strains Isolated From Korean Patients.” Annals of Clinical Microbiology and Antimicrobials 20, no. 1: 77. 34758824 10.1186/s12941-021-00481-4 PMC8579644

[mbo370074-bib-0032] Islam, M. A. , M. R. Islam , R. Khan , et al. 2022. “Prevalence, Etiology and Antibiotic Resistance Patterns of Community‐Acquired Urinary Tract Infections in Dhaka, Bangladesh.” PLoS One 17, no. 9: e0274423. 36107878 10.1371/journal.pone.0274423 PMC9477272

[mbo370074-bib-0033] Jia, P. , Y. Zhu , X. Li , et al. 2021. “High Prevalence of Extended‐Spectrum Beta‐Lactamases in *Escherichia coli* Strains Collected From Strictly Defined Community‐Acquired Urinary Tract Infections in Adults in China: A Multicenter Prospective Clinical Microbiological and Molecular Study.” Frontiers in Microbiology 12: 663033. 34305831 10.3389/fmicb.2021.663033 PMC8292957

[mbo370074-bib-0034] Jiménez Pearson, M. A. , M. Galas , A. Corso , et al. 2019. “Consenso Latinoamericano Para Definir, Categorizar Y Notificar Patógenos Multirresistentes, Con Resistencia Extendida O Panresistentes.” Revista Panamericana de Salud Pública 43: e65. 31456820 10.26633/RPSP.2019.65 PMC6705331

[mbo370074-bib-0035] Johnson, J. R. , and A. L. Stell . 2000. “Extended Virulence Genotypes of *Escherichia coli* Strains From Patients With Urosepsis in Relation to Phylogeny and Host Compromise.” Journal of Infectious Diseases 181, no. 1: 261–272. 10608775 10.1086/315217

[mbo370074-bib-0036] Khairy, R. M. , E. S. Mohamed , H. M. Abdel Ghany , and S. S. Abdelrahim . 2019. “Phylogenic Classification and Virulence Genes Profiles of Uropathogenic *E. coli* and Diarrhegenic *E. coli* Strains Isolated From Community Acquired Infections.” PLoS One 14, no. 9: e0222441. 31513642 10.1371/journal.pone.0222441 PMC6742363

[mbo370074-bib-0037] Kumar, N. , K. Chatterjee , S. Deka , R. Shankar , and D. Kalita . 2021. “Increased Isolation of Extended‐Spectrum Beta‐Lactamase‐Producing *Escherichia coli* From Community‐Onset Urinary Tract Infection Cases in Uttarakhand, India.” Cureus 13, no. 3. 10.7759/cureus.13837 PMC8036173 33854854

[mbo370074-bib-0038] Lara, F. B. , D. R. Nery , P. M. de Oliveira , et al. 2017. “Virulence Markers and Phylogenetic Analysis of *Escherichia coli* Strains With Hybrid EAEC/UPEC Genotypes Recovered From Sporadic Cases of Extraintestinal Infections.” Frontiers in Microbiology 8: 146. 28217123 10.3389/fmicb.2017.00146 PMC5290387

[mbo370074-bib-0039] Magiorakos, A. P. , A. Srinivasan , R. B. Carey , et al. 2012. “Multidrug‐Resistant, Extensively Drug‐Resistant and Pandrug‐Resistant Bacteria: An International Expert Proposal for Interim Standard Definitions for Acquired Resistance.” Clinical Microbiology and Infection 18, no. 3: 268–281. 21793988 10.1111/j.1469-0691.2011.03570.x

[mbo370074-bib-0040] Manges, A. R. , H. M. Geum , A. Guo , T. J. Edens , C. D. Fibke , and J. D. D. Pitout . 2019. “Global Extraintestinal Pathogenic *Escherichia coli* (EXPEC) Lineages.” Clinical Microbiology Reviews 32, no. 3: e00135‐18. 31189557 10.1128/CMR.00135-18 PMC6589867

[mbo370074-bib-0041] Matsukawa, M. , M. Igarashi , H. Watanabe , et al. 2019. “Epidemiology and Genotypic Characterisation of Dissemination Patterns of Uropathogenic *Escherichia coli* in a Community.” Epidemiology and Infection 147: e148. 30869058 10.1017/S0950268819000426 PMC6518783

[mbo370074-bib-0042] Miranda‐Estrada, L. I. , M. Ruíz‐Rosas , J. Molina‐López , I. Parra‐Rojas , E. González‐Villalobos , and N. Castro‐Alarcón . 2017. “Relación Entre Factores De Virulencia, Resistencia a Antibióticos Y Los Grupos Filogenéticos De *Escherichia coli* Uropatógena En Dos Localidades De México.” Enfermedades Infecciosas y Microbiología Clínica 35, no. 7: 426–433. 27048964 10.1016/j.eimc.2016.02.021

[mbo370074-bib-0043] Naghavi, M. , S. E. Vollset , K. S. Ikuta , et al. 2024. “Global Burden of Bacterial Antimicrobial Resistance 1990–2021: A Systematic Analysis With Forecasts to 2050.” The Lancet 404, no. 10459: 1199–1226. https://linkinghub.elsevier.com/retrieve/pii/S0140673624018671. 10.1016/S0140-6736(24)01867-1 PMC11718157 39299261

[mbo370074-bib-0044] Nascimento, J. A. S. , F. F. Santos , J. F. Santos‐Neto , et al. 2022. “Molecular Epidemiology and Presence of Hybrid Pathogenic *Escherichia coli* Among Isolates From Community‐Acquired Urinary Tract Infection.” Microorganisms 10, no. 2: 302. 35208757 10.3390/microorganisms10020302 PMC8874565

[mbo370074-bib-0045] Pagani, L. , E. Dell'Amico , R. Migliavacca , et al. 2003. “Multiple CTX‐M‐Type Extended‐Spectrum β‐Lactamases in Nosocomial Isolates of *Enterobacteriaceae* From a Hospital in Northern Italy.” Journal of Clinical Microbiology 41, no. 9: 4264–4269. 12958255 10.1128/JCM.41.9.4264-4269.2003 PMC193787

[mbo370074-bib-0046] Pitout, J. D. D. , G. Peirano , L. Chen , R. DeVinney , and Y. Matsumura . 2022. “ *Escherichia coli* ST1193: Following in the Footsteps of *E. coli* ST131.” Antimicrobial Agents and Chemotherapy 66, no. 7: e00511‐22. 35658504 10.1128/aac.00511-22 PMC9295538

[mbo370074-bib-0047] Poey, M. E. , M. Albini , G. Saona , and M. Laviña . 2012. “Virulence Profiles in Uropathogenic Escherichia coli Isolated From Pregnant Women and Children With Urinary Tract Abnormalities.” Microbial Pathogenesis 52, no. 5: 292–301. 22406645 10.1016/j.micpath.2012.02.006

[mbo370074-bib-0048] Rezatofighi, S. E. , M. Mirzarazi , and M. Salehi . 2021. “Virulence Genes and Phylogenetic Groups of Uropathogenic *Escherichia coli* Isolates From Patients With Urinary Tract Infection and Uninfected Control Subjects: A Case‐Control Study.” BMC Infectious Diseases 21, no. 1: 361. 33865334 10.1186/s12879-021-06036-4 PMC8052790

[mbo370074-bib-0049] Sabaté, M. , E. Moreno , T. Pérez , A. Andreu , and G. Prats . 2006. “Pathogenicity Island Markers in Commensal and Uropathogenic *Escherichia coli* Isolates.” Clinical Microbiology and Infection 12, no. 9: 880–886. 16882293 10.1111/j.1469-0691.2006.01461.x

[mbo370074-bib-0050] Sati, H. , E. Carrara , A. Savoldi , et al. 2025. “The WHO Bacterial Priority Pathogens List 2024: A Prioritisation Study to Guide Research, Development, and Public Health Strategies Against Antimicrobial Resistance.” Lancet Infectious Diseases 25, no. 9: 1033–1043. 40245910 10.1016/S1473-3099(25)00118-5 PMC12367593

[mbo370074-bib-0051] De Souza, G. M. , E. R. Dos Santos Neto , A. Martins da Silva , et al. 2019. “Comparative Study of Genetic Diversity, Virulence Genotype, Biofilm Formation and Antimicrobial Resistance Of Uropathogenic *Escherichia coli* (UPEC) Isolated From Nosocomial and Community Acquired Urinary Tract Infections.” Infection and Drug Resistance 12: 3595–3606. 31819543 10.2147/IDR.S228612 PMC6878931

[mbo370074-bib-0052] de Souza da‐Silva, A. P. , V. S. de Sousa , L. G. de Araújo Longo , et al. 2020. “Prevalence of Fluoroquinolone‐Resistant and Broad‐Spectrum Cephalosporin‐Resistant Community‐Acquired Urinary Tract Infections in Rio De Janeiro: Impact of Genotypes ST69 and ST131.” Infection, Genetics and Evolution 85: 104452. 10.1016/j.meegid.2020.104452 32634601

[mbo370074-bib-0053] Mostaghimi, T. , A. Pournajaf , A. Bijani , M. Mohammadi , M. Rajabnia , and M. Halaji . 2024. “Phylogenetic Analysis, Biofilm Formation, Antimicrobial Resistance and Relationship Between These Characteristics in Uropathogenic *Escherichia coli* .” Molecular Biology Reports 51, no. 1: 327. 38393446 10.1007/s11033-023-09031-x

[mbo370074-bib-0054] Tano, Z. N. , R. K. Kobayashi , E. P. Candido , et al. 2022. “Susceptibility to First Choice Antimicrobial Treatment for Urinary Tract Infections to *Escherichia coli* Isolates From Women Urine Samples in Community South Brazil.” Brazilian Journal of Infectious Diseases 26, no. 3: 102366. 10.1016/j.bjid.2022.102366 PMC9217753 35594950

[mbo370074-bib-0055] Tsui, C. K. , F. Ben Abid , C. L. McElheny , et al. 2024. “Characterization of *Bla* NDM in Two *Escherichia coli* ST1193 Clinical Isolates in the Gulf Region.” JAC‐Antimicrobial Resistance 6, no. 6: 166. 10.1093/jacamr/dlae166 PMC11538964 39507943

[mbo370074-bib-0056] Venugopal, S. , S. Chunchanur , R. Panigrahy , et al. 2024. “Changes in Antimicrobial Resistance of *Escherichia coli* Isolated From Community‐Associated Urinary Tract Infection before and During the COVID‐19 Pandemic in India.” Journal of Global Antimicrobial Resistance 37: 165–167. 38458537 10.1016/j.jgar.2024.02.022

[mbo370074-bib-0057] Wang, M. C. , Y. H. Fan , Y. Z. Zhang , et al. 2023. “Characterization of Uropathogenic *Escherichia coli* Phylogroups Associated With Antimicrobial Resistance, Virulence Factor Distribution, and Virulence‐Related Phenotypes.” Infection, Genetics and Evolution 114: 105493. 10.1016/j.meegid.2023.105493 37634856

[mbo370074-bib-0058] Wirth, T. , D. Falush , R. Lan , et al. 2006. “Sex and Virulence in *Escherichia coli*: An Evolutionary Perspective.” Molecular Microbiology 60, no. 5: 1136–1151. 16689791 10.1111/j.1365-2958.2006.05172.x PMC1557465

[mbo370074-bib-0059] Yamaji, R. , J. Rubin , E. Thys , C. R. Friedman , and L. W. Riley . 2018. “Persistent Pandemic Lineages of Uropathogenic *Escherichia coli* in a College Community From 1999 to 2017.” Journal of Clinical Microbiology 56, no. 4: e01834‐17. 29436416 10.1128/JCM.01834-17 PMC5869836

[mbo370074-bib-0060] Yamamoto, S. , A. Terai , K. Yuri , H. Kurazono , Y. Takeda , and O. Yoshida . 1995. “Detection of Urovirulence Factors in Escherichia coli by Multiplex Polymerase Chain Reaction.” FEMS Immunology and Medical Microbiology 12, no. 2: 85–90. 8589667 10.1111/j.1574-695X.1995.tb00179.x

[mbo370074-bib-0061] Yazdanpour, Z. , O. Tadjrobehkar , and M. Shahkhah . 2020. “Significant Association Between Genes Encoding Virulence Factors With Antibiotic Resistance and Phylogenetic Groups in Community Acquired Uropathogenic *Escherichia coli* Isolates.” BMC Microbiology 20, no. 1: 241. 32758126 10.1186/s12866-020-01933-1 PMC7409443

[mbo370074-bib-0062] Zeng, Q. , S. Xiao , F. Gu , et al. 2021. “Antimicrobial Resistance and Molecular Epidemiology of Uropathogenic *Escherichia coli* Isolated From Female Patients in Shanghai, China.” Frontiers in Cellular and Infection Microbiology 11: 653983. 34485168 10.3389/fcimb.2021.653983 PMC8414883

[mbo370074-bib-0063] Zeng, Z. , J. Zhan , K. Zhang , H. Chen , and S. Cheng . 2022. “Global, Regional, and National Burden of Urinary Tract Infections From 1990 to 2019: An Analysis of the Global Burden of Disease Study 2019.” World Journal of Urology 40, no. 3: 755–763. 35066637 10.1007/s00345-021-03913-0

