# Supplementary material for: High Rates of Antimicrobial Resistance and Emergence of High‐Risk Clones in Community‐Acquired Uropathogenic Escherichia coli
Source: Microbiologyopen. 2025 Oct 21;14(5):e70074. doi: 10.1002/mbo3.70074 (PMC12540921; doi:10.1002/mbo3.70074)
Supplement: Supplementary file 1 — Table A1: Oligonucleotides for the identification of virulence factors and pathogenicity islands. Table A2: Virulence factors and islands of pathogenicity relation with phylogenetic groups of E. coli isolates from CA‐UTI (n=98). Table A3: Patterns of virulence genes in E. coli from community‐acquired urinary tract infections, stratified by phylogenetic groups. [file MBO3-14-e70074-s001.docx]

**Table A1**: Oligonucleotides for the identification of virulence factors and pathogenicity islands.

| **Genes** | **Primer sequences (5’ → 3’)** | **Amplicon size (bp)** | | **Annealing**  **(ºC)** | **Reference** |
| --- | --- | --- | --- | --- | --- |
| ***hlyA*-f** | AACAAGGATAAGCACTGTTCTGGCT | | 1177 | 63 | (62) |
| ***hlyA*-r** | ACCATATAAGCGGTCATTCCCGTCA | |  |  |  |
| ***aer*-f** | TACCGGATTGTCATATGCAGACCGT | | 602 | 63 | (62) |
| ***aer*-r** | AATATCTTCCTCCAGTCCGGAGAAG | |  |  |  |
| ***cnf-1*-f** | AAGATGGAGTTTCCTATGCAGGAG | | 498 | 56 | (62) |
| ***cnf-1*-r** | CATTCAGAGTCCTGCCCTCATTATT | |  |  |  |
| ***papC*-f** | GACGGCTGTACTGCAGGGTGTGGCG | | 328 | 65 | (62) |
| ***papC*-r** | ATATCCTTTCTGCAGGGATGCAATA | |  |  |  |
| ***sfa*-f** | CTCCGGAGAACTGGGTGCATCTTAC | | 410 | 67 | (62) |
| ***sfa*-r** | CGGAGGAGTAATTACAAACCTGGCA | |  |  |  |
| ***afa*-f** | GCTGGGCAGCAAACTGATAACTCTC | | 750 | 63 | (62) |
| **afa-r** | CATCAAGCTGTTTGTTCGTCCGCCG | |  |  |  |
| ***fimH*-f** | TTTTGCGACAGACCAACAACTAT | | 419 | 55 | (63) |
| ***fimH*-r** | TTGCACATTCCCTGCAGTCAC | |  |  |  |
| ***papG*-f** | CATTTATCGTCCTCAACTTAG | | 482 | 55 | (62) |
| ***papG*-r** | AAGAAGGGATTTTGTAGCGTC | |  |  |  |
| ***draD*-f** | ATGAACGGGAGTATAAGGAAGA | | 407 | 52 | (63) |
| ***draD*-r** | AACCGGTATTCACCAGGAGCAA | |  |  |  |
| ***iutA*-f** | GGCTGGACATCATGGGAACTGG | | 300 | 63 | (23) |
| ***iutA*-r** | CGTCGGGAACGGGTAGAATCG | |  |  |  |
| ***traT*-f** | GGTGTGGTGCGATGAGCACAG | | 290 | 63 | (23) |
| ***traT*-r** | CACGGTTCAGCCATCCCTGAG | |  |  |  |
| ***neuA*-f** | CTTCACCCTTTAGAGATTCGACCC | | 474 | 55 | (63) |
| ***neuA*-r** | CCCAATAATCAAACAGCGAGTGTCC | |  |  |  |
| ***kpsM*-f** | GCGCATTTGCTGATACTGTTGGG | | 454 | 55 | (63) |
| ***kpsM*-r** | GAGGGAACATGATGCAGGAGATG | |  |  |  |
| ***iroN*-f** | AATCCGGCAAAGAGACGAACCGCCT | | 553 | 63 | (23) |
| ***iroN*-r** | GTTCGGGCAACCCCTGCTTTGACTTT | |  |  |  |
| ***fyuA*-f** | TGATTAACCCCGCGACGGGAA | | 880 | 63 | (23) |
| ***fyuA*-r** | CGCAGTAGGCACGATGTTGTA | |  |  |  |
| **PAI I_CFT073_-f** | GGACATCCTGTTACAGCGCGCA | | 930 | 55 | (23) |
| **PAI I_CFT073_-r** | TCGCCACCAATCACAGCGAAC | |  |  |  |
| **PAI II_CFT073_-f** | ATGGATGTTGTATCGCGC | | 400 | 55 | (24) |
| **PAI II_CFT073_-r** | ACGAGCATGTGGATCTGC | |  |  |  |
| **PAI IV _536_-f** | AAGGATTCGCTGTTACCGGAC | | 300 | 55 | (24) |
| **PAI IV_536_-r** | TCGTCGGGCAGCGTTTCTTCT | |  |  |  |

**Table A2**: Virulence factors and islands of pathogenicity relation with phylogenetic groups of *E. coli* isolates from CA-UTI (n=98).

| **Trait** | **Prevalence of traits (no. of isolates [%])** | | ***p* value^a^** | **Univariate** | | **Multivariate^b^** | |
| --- | --- | --- | --- | --- | --- | --- | --- |
|  | **B2 group (n=47)** | **Non-B2 (n=51)** |  | **OR** | **95% CI** | **OR** | **95% CI** |
| ***papG*** | 5 (10.6) | 0 | **0.02** | 37.1 | 0.24-5.62 |  |  |
| ***cnf1*** | 8 (17.0) | 0 | **<0.01** | 63.0 | 0.46-8.72 |  |  |
| ***sfa*** | 12 (25.5) | 0 | **<0.01** | 104 | 0.80-13.68 |  |  |
| ***hlyA*** | 12 (25.5) | 1 (2.0) | **<0.01** | 14.7 | 2.06-104 |  |  |
| *draD/afa* | 1 (2.1) | 3 (5.9) | 0.61 | 0.39 | 0.04-3.64 |  |  |
| *iroN* | 13 (27.7) | 6 (11.8) | 0.07 | 2.80 | 0.97-8.10 |  |  |
| *papC* | 12 (25.5) | 9 (17.6) | 0.46 | 1.59 | 0.60-4.20 |  |  |
| *traT* | 11 (23.4) | 17 33.3) | 0.37 | 0.62 | 0.25-1.50 |  |  |
| ***neuA*** | 21 (44.7) | 9 (17.6) | **<0.01** | 3.69 | 1.47-9.24 | 12.00 | 3.52–51.92 |
| *iutA* | 21 (44.7) | 23 45.1) | >0.99 | 1.36 | 0.61-3.03 |  |  |
| *aer* | 23 (48.9) | 22 43.1) | 0.68 | 1.26 | 0.57-2.79 |  |  |
| ***kpsMT*** | 43 (91.5) | 13 25.5) | **<0.01** | 29.1 | 8.88-95.3 |  |  |
| ***fyuA*** | 46 (97.9) | 29 56.9) | **<0.01** | 29.5 | 4.27-204 |  |  |
| *fimH* | 47 (100) | 47 92.2) | 0.12 | 24.7 | 0.15-4.10 |  |  |
| **PAI I_CFT073_** | 18 (38.3) | 3 (5.9) | **<0.01** | 9.33 | 2.59-33.6 |  |  |
| **PAI II_CFT073_** | 27 (57.4) | 2 (3.9) | **<0.01** | 29.8 | 6.84-130 | 59.02 | 12.80–458.75 |
| **PAI IV_536_** | 46 (97.9) | 30 (58.8) | **<0.01** | 27.2 | 3.94-188 | 46.76 | 3.58–2105.92 |

^a^Fisher’s exact test. ^b^R^2^ Tjur 0.583; ^b^AIC 73.754

**Table A3**: Patterns of virulence genes in *E. coli* from community-acquired urinary tract infections, stratified by phylogenetic groups.

| Phylogroup | Virulence genes | Number of isolates  n=98 | | |
| --- | --- | --- | --- | --- |
| A | *fimH* | 2 | | |
|  | *fimH, traT* | | 1 |  |
|  | *fimH, aer*, PAI IV_536_ | | 1 |  |
|  | *fyuA,* PAI I _CFT073_, PAI IV_536_ | | 1 |  |
|  | *fimH, fyuA,* PAI IV_536_ | | 1 |  |
|  | *fimH, fyuA, aer,* PAI IV_536_ | | 1 |  |
|  | *fimH, fyuA, traT,* PAI IV_536_ | | 1 |  |
|  | *fimH, neuA, traT, iroN* | | 1 |  |
|  | *fimH, aer, iutA, traT, iroN* | | 2 |  |
|  | *fimH, fyuA, aer, iutA, traT, draD/afa,* PAI IV_536_ | | 1 |  |
|  | *fimH, fyuA, aer, ituA, traT, draD/afa, kpsM,* PAI IV_536_ | | 1 |  |
| B1 | *fimH* | | 5 |  |
|  | *fimH, aer* | | 2 |  |
|  | *fyuA, papC* | | 1 |  |
|  | *fimH, traT* | | 1 |  |
|  | *fimH, neuA* | | 1 |  |
|  | *fimH,* PAI IV_536_ | | 1 |  |
|  | *fimH, aer, iutA* | | 1 |  |
|  | *fimH, fyuA,* PAI IV_536_ | | 4 |  |
|  | *fimH, fyuA, traT,* PAI IV_536_ | | 2 |  |
| B2 | *fimH, fyuA,* PAI IV_536_ | | 1 |  |
|  | *fimH, fyuA, neuA, kpsM,* PAI II_CFT073_ | | 1 |  |
|  | *fimH, fyuA, neuA, kpsM,* PAI IV_536_ | | 1 |  |
|  | *fimH, fyuA, traT, kpsM,* PAI IV_536_ | | 1 |  |
|  | *fimH, fyuA, kpsM, aer, iutA,* PAI IV_536_ | | 2 |  |
|  | *fimH, fuyA, kpsM,* PAI I _CFT073_, PAI II _CFT073_, PAI IV_536_ | | 5 |  |
|  | *fimH, fyuA, papC, draD/afa,* PAI IV_536_ | | 1 |  |
|  | *fimH, fyuA, traT,* PAI I _CFT073_, PAI II _CFT073_*,* PAI IV_536_ | | 1 |  |
|  | *fimH, fyuA, kpsM,* *neuA, traT,* PAI IV_536_ | | 1 |  |
|  | *fimH, fyuA, kpsM, aer, iutA, neuA,* PAI IV_536_ | | 13 |  |
|  | *fimH, fyuA, kpsM, neuA,* PAI I _CFT073_, PAI II _CFT073_, PAI IV_536_ | | 1 |  |
|  | *fimH, fyuA, kpsM, neuA, iroN, sfa,* PAI II _CFT073_, PAI IV_536_ | | 1 |  |
|  | *fimH, fyuA, aer, iutA, papC,* PAI I _CFT073_, PAI II _CFT073_, PAI IV_536_ | | 1 |  |
|  | *fimH, kpsM, aer, iutA, traT,* PAI I _CFT073_, PAI II _CFT073_, PAI IV_536_ | | 1 |  |
|  | *fimH, fyuA, kpsM, iroN, sfa,* PAI I _CFT073_, PAI II _CFT073_, PAI IV_536_ | | 1 |  |
|  | *fimH, fyuA, kpsM, aer, iutA, neuA,* PAI II _CFT073_, PAI IV_536_ | | 1 |  |
|  | *fimH, fyuA, kpsM, neuA, iroN, sfa,* PAI I _CFT073_, PAI II _CFT073_, PAI IV_536_ | | 1 |  |
|  | *fimH, fyuA, kpsM, hlyA, cnf-1, papC, papG,* PAI II _CFT073_, PAI IV_536_ | | 1 |  |
|  | *fimH, fyuA, kpsM, hlyA, traT, cnf-1, papC, papG,* PAI II _CFT073_, PAI IV_536_ | | 2 |  |
|  | *fimH, fyuA, kpsM, iroN, cnf-1, hlyA, sfa,* PAI I _CFT073_, PAI II _CFT073_, PAI IV_536_ | | 1 |  |
|  | *fimH, fyuA, kpsM, aer, papC, iroN, hlyA, sfa,* PAI I _CFT073_, PAI II _CFT073_, PAI IV_536_ | | 1 |  |
|  | *fimH, fyuA, kpsM, traT, iroN, hlyA, sfa, cnf-1,* PAI I _CFT073_, PAI II _CFT073_, PAI IV_536_ | | 2 |  |
|  | *fimH, fyuA, kpsM, aer, iutA, papC, iroN, hlyA, sfa,* PAI II _CFT073_, PAI IV_536_ | | 1 |  |
|  | *fimH, fyuA, kpsM, papC, iroN, hlyA, sfa, cnf-1, papG,* PAI II _CFT073_, PAI IV_536_ | | 1 |  |
|  | *fimH, fyuA, kpsM, aer, iutA, neuA, traT, papC, iroN,* PAI I _CFT073_, PAI II _CFT073_, PAI IV_536_ | | 1 |  |
|  | *fimH, fyuA, kpsM, traT, papC, iroN, hlyA, sfa, cnf-1, papG,* PAI II _CFT073_, PAI IV_536_ | | 1 |  |
|  | *fimH, fyuA, kpsM, aer, traT, papC, iroN, hlyA, sfa,* PAI I _CFT073_, PAI II _CFT073_, PAI IV_536_ | | 1 |  |
|  | *fimH, fyuA, kpsM, aer, iutA, papC, iroN, hlyA, sfa,* PAI I _CFT073_, PAI II _CFT073_, PAI IV_536_ | | 1 |  |
| C | *fimH, fyuA, neuA,* PAI IV_536_ | | 1 |  |
|  | *fimH, aer, iutA, traT, iroN* | | 1 |  |
| D | *fimH, kpsM* | | 1 |  |
|  | *fyuA, iutA,* PAI IV_536_ | | 1 |  |
|  | *fyuA, kpsM, aer, papC,* PAI IV_536_ | | 1 |  |
|  | *fimH, fyuA, kpsM, neuA,* PAI IV_536_ | | 1 |  |
|  | *fimH, fyuA, papC, iroN, hlyA,* PAI IV_536_ | | 1 |  |
|  | *fimH, fyuA, kpsM, aer, iutA,* PAI II _CFT073_*,* PAI IV_536_ | | 1 |  |
|  | *fimH, fyuA, kpsM, aer, iutA, traT, iroN,* PAI IV_536_ | | 1 |  |
|  | *fimH, fyuA, kpsM, aer, iutA, draD/afa,* PAI II _CFT073_*,* PAI IV_536_ | | 1 |  |
|  | *fimH, fyuA, kpsM, aer, iutA, traT,* PAI IV_536_ | | 2 |  |
| E | *fimH* | | 1 |  |
|  | *fimH, neuA* | | 1 |  |
| F | *fimH, fyuA, kpsM, aer, iutA, neuA, papC,* PAI IV_536_ | | 2 |  |
|  | *fimH, fyuA, kpsM, aer, iutA, neuA, papC,* PAI I _CFT073_, PAI IV_536_ | | 1 |  |
|  | *fimH, fyuA, kpsM, aer, traT, neuA, papC,* PAI I _CFT073_, PAI IV_536_ | | 1 |  |
| NT | *fimH, fyuA, aer, iutA, traT, papC,* PAI IV_536_ | | 2 |  |
